# Recombinant *Vaccinia virus*-coded interferon inhibitor B18R: Expression, refolding and a use in a mammalian expression system with a RNA-vector

**DOI:** 10.1371/journal.pone.0189308

**Published:** 2017-12-07

**Authors:** Yuriy G. Kim, Aliya Zh. Baltabekova, Erzhan E. Zhiyenbay, Altynai S. Aksambayeva, Zhadyra S. Shagyrova, Rinat Khannanov, Erlan M. Ramanculov, Alexandr V. Shustov

**Affiliations:** 1 National Laboratory Astana, Nazarbayev University, Astana, Kazakhstan; 2 National Center for Biotechnology, Astana, Kazakhstan; Weizmann Institute of Science, ISRAEL

## Abstract

B18R protein of *Vaccinia virus* binds to type I interferons and inhibits activation of interferon-mediated signal transduction. Cells which have unimpaired interferon signaling such as primary cell cultures or some industrially important cell lines are capable of development of an antiviral state. An establishment of the antiviral state limits replication of RNA-viruses and can suppress replication of RNA vectors. The interferon inhibitor B18R effectively prevents the establishment of the antiviral state. For this reason, B18R has become a ubiquitous component of protocols for epigenetic reprogramming which use transfections of RNA replicons or mRNA. Despite wide practical applicability, commercially available B18R is predominantly produced in cell cultures and little information has been published on a production and use of bacterially expressed B18R. Objectives of this study were to produce B18R in an *E*.*coli* expression system and to confirm the product’s biological activity by using it to maintain RNA-vectors in cell cultures capable of the antiviral state. The described method allows the expression and efficient refolding to obtain 10–100 mg of B18R from a small-scale culture and the production process is economically attractive compared to a use of an eukaryotic expression. To check for a presence of the biological activity of bacterially-expressed B18R the protein was used to support persistence of an autonomously replicating RNA-vector in a cell culture which is capable of the antiviral state. A RNA-containing virus, Venezuelan equine encephalitis virus (VEE) can serve as an efficient vector for heterologous expression in cell cultures, although its replication is sensitive to the effects of type I interferons which limit a range of cell lines for a use with this vector. The VEE replicon was utilized to direct an expression of recombinant human granulocyte colony stimulating factor (G-CSF). The producing replicon could persist in HEK293 cells for sufficiently long time only in presence of B18R, whereas addition of B18R not only allowed persistence of the replicon but also increased production from the replicon. A model product granulocyte colony stimulating factor accumulated to 35.5 μg/ml during a 7 day experiment. This work describes efficacious expression and refolding of the viral cytokine inhibitor and demonstrates a utility of bacterially-expressed B18R.

## Introduction

Various types of eukaryotic vectors have been developed to engineer a recombinant expression in cell cultures. Expression vectors based on genomes of viruses or autonomously replicating fragments of virus genomes (replicons) are a kind of vectors which currently undergo transition from experimental efforts to industrial routines. The most popular virus backbones used as vectors in mammalian cell cultures (i.e., excluding applications in gene therapy) are adenoviruses [1], retroviruses [2] and in particular lentiviruses [3], poxviruses (*Vaccinia virus*) [4] and modified baculoviruses (viruses of insects) engineered to express mammalian gene cassettes [5]. All these vectors are either DNA viruses (i.e., they have a DNA genome), or viruses which have obligatory reverse transcription in their life cycles which results in a generation of DNA-copies of the genomes (proviruses), and the proviruses are integrated into a host’s genome. A different type of a virus vector is represented by RNA-viruses which do not use reverse transcription.

Alphaviruses which are representatives of the genus Alphavirus, family Togaviridae, are prominent vectors for heterologous expression [6]. There are 35 species which are grouped into subgroups in accordance with their geographic prevalence and named after their most studied members, e.g. the *Semliki Forest virus* (SFV) subgroup, *Sindbis virus* (SIN) subgroup and *Venezuelan equine encephalitis virus* (VEE) subgroup. Utilized vectors include SFV [7, 8], SIN [9] and VEE [10, 11]. A replication of the alphavirus’ genome is accompanied by a generation of an impressive amount of proteins encoded in a virus’ subgenomic RNA (sgRNA); this is because within few hours upon entry into a cell the sgRNA becomes the predominant type of mRNA. This leads to the synthesis of recombinant proteins up to 25% of the total cellular protein [12]. Utilization of alphaviruses as vectors faces a problem of inhibition of a replication by an innate immune response which is conferred by interferons (IFNs). IFNs are a heterogeneous family of secreted glycoproteins which include several types. The type I IFNs includes IFN-alpha, -beta, -delta and -kappa [13, 14], type II is IFN-gamma, and type III is IFN-lamda. IFNs exert their regulation through an interaction with type-specific receptors on cell membranes (IFNAR for the type I IFN, IFNGR for type II, IL-28 R alpha/IL-10 R beta for type III). IFNs signal through JAK-STAT pathway, upregulate many interferon-stimulated genes (ISG) and induce a multitude of intracellular factors, among which are the protein kinase PKR, 2’,5’-oligoadenylate synthetase, RNase L and the GTPases of Mx proteins. Ultimate consequences of the IFN-induced regulation include degradation of double-stranded RNAs (the replicative intermediate of RNA-viruses) and inhibition of protein synthesis.

Viruses of different genera and even different species within a same genus vary in their sensitivity to the IFNs. Alphaviruses are sensitive to the type I IFNs [15, 16, 17, 18]. Also, autonomously replicating fragments of genomes (replicons) of alphaviruses show various degrees of IFN-resistance and induce the antiviral state as seen with homologous alphaviruses [19].

Supplementation of cultures with inhibitors of IFNs may provide for a replication of the alphavirus vectors in cells which have unimpaired IFN regulation (such as primary cell cultures) and also widen a scope of possible applications of these vectors. In this regard a protein B18R attracts a special interest. Poxviruses encode several proteins which intercept cytokines and B18R works as an inhibitor of the type I IFNs.

B18R is encoded by Western Reserve (WR) strain of *Vaccinia virus*. B18R works as a decoy receptor for the type I IFNs, attaches to cell surfaces, binds IFNs [20] without prominent species-specificity [21] and prevents an establishment of the antiviral state [22]. At a same time B18R has no effect on cell viability itself [23]. Thereunder B18R has become an essential aid in cell technologies which use RNA-mediated gene delivery [11, 24]. An exemplary work [11] describes a use of alphavirus replicons as vectors to produce pluripotency factors to direct reprogramming of human fibroblasts into induced pluripotent stem cells (IPSC). Addition of B18R to primary cell cultures was required because it was shown that without an inhibition of the IFN-response the VEE replicons cannot infect the primary cultures [11]. A utility of B18R goes beyond the maintenance of RNA-replicons; a presence of B18R also increases a viability of cultures upon transfections with synthetic mRNAs [25].

Despite an established high demand for this protein in cell technologies and also attractive prospects for use in mammalian expression systems to maintain RNA-vectors, little information is available on obtaining of B18R. In majority of works utilizing it, this protein has not been produced in a pure form. Rather, conditioned media (CM) are collected from cell cultures infected with *Vaccinia virus* [26, 22, 27] or transfected with a B18R mRNA [11]. The CM containing secretory-expressed B18R are used without an isolation of B18R. As the production in cell cultures is expensive, a development of a bacterial expression system is warranted, although to date only one paper describes the expression/refolding of B18R in *E*.*coli* [28] and the published protocol is brief and difficult to reproduce. In this paper we describe the bacterial expression and a different method for refolding of recombinant B18R. Multi-milligram quantities of recombinant B18R are easily produced and the product is biologically active despite that bacterially-expressed B18R is not glycosylated. It has been successfully utilized to support persistence of a RNA vector derived from a genome of a RNA-virus in cell cultures capable of the antiviral state. In the presence of B18R a remarkably higher amount of a model biopharmaceutical protein was produced. Thus, described B18R is a useful addition in a development of mammalian expression systems with RNA-vectors.

## Materials and methods

Detailed protocol for isolation and refolding of B18R is submitted to protocols.io with the DOI: 10.17504/protocols.io.kp8cvrw.

### Synthesis of genes

Synthetic genes (B18R, G-CSF) and DNA fragments used to assemble VEE replicons were produced from oligonucleotides using an overlap extension PCR [29]. All synthesizedDNA fragments were cloned in a pGEM-T vector (Promega) and sequencing-verified. Resulting clones were used to assemble constructs described herein.

### Production of recombinant B18R

**Fig 1:**
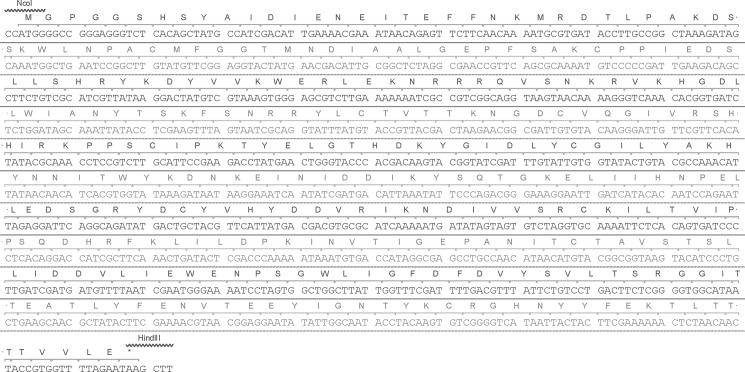
Part of the expression plasmid with a synthetic gene B18R. The deduced translation of recombinant B18R is shown.

A gene was synthesized which encodes the aminoacid residues His20-Glu351 from a B18R protein of the Western Reserve strain of *Vaccinia virus* (Genbank D01019). The gene B18R has a nucleotide sequence which is codon-optimized for an expression in *E*.*coli*. A vector pET28c (Novagen) was used to construct an expression plasmid pET28-B18R(HisTag) which sequence is deposited to the Genbank entry MG356786. The expression product has a hexahistidine tag at the N-terminus. The plasmid’s fragment with the B18R gene is depicted in Fig 1.

Cells of a *E*.*coli* strain BL21(DE3) were transformed with the plasmid pET28-B18R(HisTag). The expression strain was inoculated into 1L of Luria-Bertani (LB) medium with kanamycin (50 μg/ml). The culture was incubated at 37°C on a shaker (150 rpm) until an optical density at 600 nm is 0.8. At this point isopropyl β-D-thiogalactoside (IPTG) was added to 1 mM. The induced culture was grown for 6 hrs at 37°C. Bacterial cells were collected by centrifugation. Bacterial biomass was washed in water and resuspended to obtain a 10% suspension in a lysis buffer (20% sucrose, 20 mM HEPES pH 7.5, 5 mM MgCl_2_, 0.1% Triton-X100, 1 mg/ml lysozyme, 10 μg/ml DNAse I, 100 μg/ml RNase, 0.2 mM PMSF). The suspension was incubated at room temperature for 1 hr. Then a dry sodium deoxycholate (SDC) was added to the suspension to 0.5% (w/v). Completeness of lysis was achieved by sonication (20 pulses, each pulse 30 sec, with 3 min pauses). The insoluble fraction which contains inclusion bodies was separated by centrifugation (12000 rpm, 30 min, 4°C). To remove bacterial lipids the pellet was washed once in a wash buffer with SDC (100 mM Tris pH 8.0, 2 mM EDTA, 0.5% SDC), and then two more times in buffer TE (100 mM Tris pH 8.0, 2 mM EDTA). Each wash step was accompanied with sonication of the suspension to disintegrate chunks of the pellet. After the last wash, the pellet was weighted and divided into portions by 70 mg, which were stored at -80°C before further processing. To extract B18R from the inclusion bodies, one portion of the pellet (70 mg) was dissolved in 100 ml of a solubilization buffer (50 mM Tris pH 10, 2% sodium lauroyl sarcosinate (SLS)). The suspensions were stirred at room temperature for 2 hours. To perform oxidation of free cysteinyl -SH groups to disulfides which is a prerequisite for refolding, CuSO_4_ was added to the protein solution to a final concentration 50 μM. Samples were stirred in an open flask on a magnetic stirrer for 20 hrs at room temperature. Upon completion of the oxidation step, an excess of the detergent was removed. For this purpose the protein was precipitated from the solution. EDTA was added to 10 mM to chelate copper ions and then dry ammonium sulfate (25 g) was slowly added (final concentration 40% w/v) and the resulting solution was incubated for 2 hrs on ice. The protein was precipitated by centrifugation (10000 rpm, 20 min). The precipitate was dissolved in 20 ml of buffer A for metal-affinity chromatography (IMAC) containing 6M urea (20 mM Na_2_HPO_4_ pH 7.4, 500 mM NaCl, 20 mM imidazole, 6M urea). Undissolved material was removed by centrifugation. A first round of chromatographic purification was performed using the IMAC. A column His GraviTrap (GE Healthcare) was prepared according to the manufacturer's instructions and equilibrated with buffer A+6M urea. The B18R solution was passed through the column and the column was washed with 20 ml of buffer A+6M urea. Elution was carried out by passing through the column of 5 ml of buffer B for IMAC+6M urea (20 mM Na_2_HPO_4_ pH 7.4, 500 mM NaCl, 500 mM imidazole, 6M urea). The eluate was collected and dialyzed against 2 L of 20 mM Trizma pH9.0. A second round of purification employed anion exchange chromatography (AEX). The B18R solution from a dialysis bag was slowly loaded onto HiTrap Q HP 5 ml column (GE Healthcare) prepared in accordance to the manufacturer’s instructions and balanced with buffer A for AEX (20 mM Tris-HCl pH9.0). The column was washed with 25 ml of buffer A (AEX). The protein was eluted by passing through the column of 15 ml of buffer B for AEX (20 mM Tris-HCl pH9.0, 1 M NaCl). The eluate from the Q-sepharose column was dialyzed against 2 L of PBS (Amresco Cat. E404) for two days at +8°C, with three buffer changes during the dialysis. The protein concentration was determined using the Bradford method. A residual *E*.*coli* lipopolysaccharide (endotoxin) was measured by the LAL method using Endosafe-PTS cartridges and a scanner (Charles River). The B18R solution was filtered through a 0.22-μm filter and stored at -80°C.

### Analytical size exclusion chromatography

An Omnifit chromatography column (150 mm × 10 mm (i.d.), Sigma Cat. 56002) was used for an analytical SEC. The column was filled with Sephacryl 200-HR (Sigma Cat. S200HR) to obtain a bed volume 9.5 ml. The column was attached to an FPLC system AKTA Purifier 10 (GE Healthcare) equipped with a 0.2 ml sample loop. To perform the assay, portions (0.2 ml) of B18R or B18R mixed with IFN-alpha 1 were loaded onto the column using the sample loop. The column was run with an HN buffer (20 mM HEPES pH7.4, 150 mM NaCl) at a rate of 2 ml/min. Absorption peaks were recorded at 280 nm to determine retention times and peak heights.

To analyze a composition of free B18R 200 μg of the protein was loaded onto the SEC column. A recombinant human IFN-alpha 1 (Sigma Cat. SRP4596) was used as a specific ligand to test an ability of B18R to bind the IFN. To prepare a complex B18R/IFN-alpha 1, 190 μg of B18R was mixed with 10 μl IFN-alpha 1 (dissolved at 2 mg/ml) and the mixture was incubated for 1 hour at room temperature before analyzing it. Obtained chromatographic profiles in numerical forms are given in Supplementary information. During the SEC of the mixture B18R+IFN-alpha 1, chromatographic peaks were collected, the collected solutions were concentrated using centrifugal concentrators Vivaspin with MWCO 5 kDa and a protein content of the concentrated samples was investigated in SDS-PAGE.

### Construction of VEE replicons

A cDNA copy of a replicon RepVEE.GFP was assembled from *de novo*-synthesized fragments (more details in Supporting information). A sequence of the replicon RepVEE.GFP is deposited to the Genbank MF136447. Its VEE-specific part corresponds to the strain TC-83 (Genbank No L01443) with one mutation (T3865->A, an encoded aminoacid changes from Gln739 to Leu in a protein nsp2). This mutation was described in [30] and was intentionally introduced into the replicon to make its replication noncytopathic. The cDNA copy was cloned in a plasmid pRepVEE.GFP wherein it is placed downstream of a promoter of SP6 RNA polymerase. The SP6 promoter allows synthesizing of the replicon RNA using an *in vitro* transcription. A unique MluI site is engineered downstream of the replicon’s 3’-end; this site is used to linearize the plasmid DNA before a run-off transcription. In the replicon RepVEE.GFP a GFP gene is engineered downstream of the first viral subgenomic promoter.

**Fig 2:**
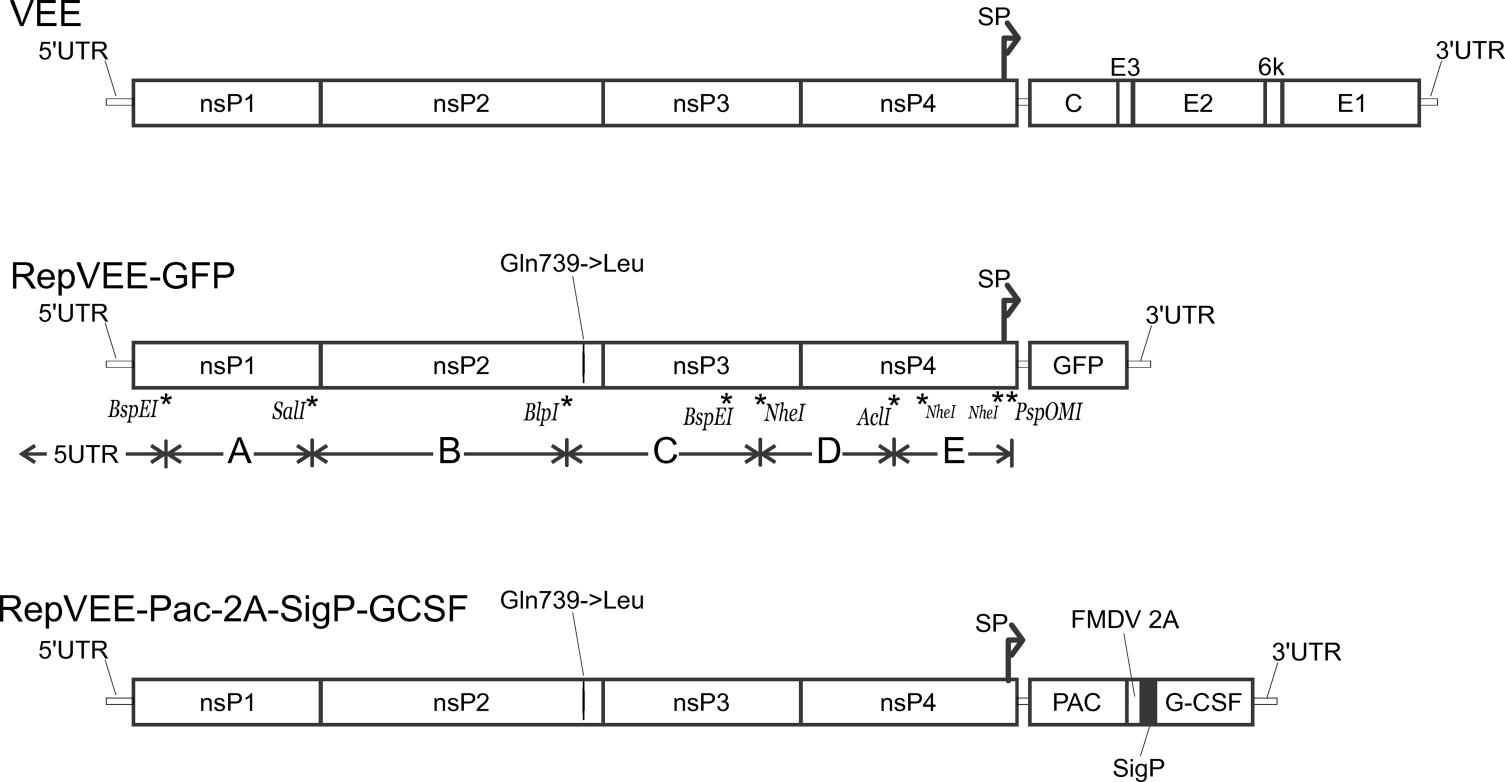
Genetic maps of the VEE genomes. Upper part: a VEE genetic map. Boxes indicate genes for individual functional proteins. Middle part: the replicon RepVEE-GFP. Positions of restriction sites used for an assembly of the replicon are indicated. Lower part: the replicon RepVEE-Pac-2A-SigP-GCSF. Designations: SP—subgenomic promoter; PAC—puromycin N-acetyltransferase; FMDV 2A - autoprotease 2A; SigP—signal peptide for a secretory expression; G-CSF—granulocyte colony stimulating factor.

A gene cassette Pac-2A-SigP-GCSF was designed to direct coexpression of an enzyme puromycin N-acetyltransferase (Pac) and a recombinant human colony stimulating factor (G-CSF). The gene cassette Pac-2A-SigP-GCSF has a following design: its single open reading frame (ORF) encodes a polyprotein wherein the N-terminal part is the Pac, the C-terminal part is the G-CSF (with a signal peptide for secretory expression, SigP) and a central region is a protease 2A of foot-and-mouth disease virus (FMDV 2A) (Fig 2). In the replicon RepVEE.Pac-2A-SP-GCSF the gene cassette is placed downstream of the subgenomic promoter. The sequence pRepVEE.Pac-2A-SP-GCSF is deposited to the Genbank MF136448.

### *In vitro* transcriptions

Plasmids were digested with MluI. Reaction mixtures for *in vitro* transcription consisted of 12.5 μl of water, 10 μl of 5X buffer for SP6 RNA polymerase, 5 μl 10 mM DTT, 2.5 μl of 10 mM synthetic cap analogue (m7G(5')ppp(5')G), 10 μl of rNTP solution (2.5 mM each rNTP), 10 μl (2 μg) of the linearized plasmid. To this mixture 1 μl of RNase inhibitor (RiboLock, ThermoFisher Scientific) and 2.5 μl of SP6 RNA polymerase (ThermoFisher Scientific) were added. The mixture was incubated at 41°C for 1 hour. Quality of RNA was examined by a gel electrophoresis in 1% agarose.

### Cell cultures and RNA transfections

BHK-21 and HEK293 cells were kindly provided by M.Saparbayev (Institut Pasteur, France). Cell cultures were grown in a complete medium composed from DMEM with high glucose (ThermoFisher Scientific) supplemented with 10% fetal bovine serum (FBS; Gibco), 1% Glutamax (Invitrogen) and a 1% mixture of penicillin/streptomycin. Cultures in 6-well plates at 70–90% confluency were transfected with the *in vitro*-synthesized RNAs by utilizing cationic liposomes (Lipofectamine 2000, Life Technologies). Where indicated, recombinant B18R was added to cultures to a working concentration 500 ng/ml. For a selection of replicon-containing cells puromycin was added to cultures to 10 μg/ml at 24 hrs after transfection.

### Producer lines and G-CSF yields

An aliquot of a replicon-containing culture was recovered from a cryo-stock and seeded into the complete medium. One day later, puromycin was added (10 μg/ml) and the cultures were grown in a presence of puromycin until 90% confluency in T150 flasks. The complete medium was removed and 50 ml of a production medium (DMEM+1% FBS, no puromycin) were added to each T150 flask. Recombinant B18R was added to producing HEK293 cultures to 500 ng/ml. The cultures were incubated for 7 days without further changes of the medium. Each day small samples (200 μl) of the culture media were collected from the flasks to determine amounts of G-CSF using a quantitative ELISA. The ELISA was done using a Human G-CSF/CSF3 ELISA Kit (Sigma Cat. RAB0103). The ELISA results are deposited to Supplementary information. Experiments were done in triplicate, averages and spreads between three data points were calculated.

## Results

### Expression and refolding of B18R

**Fig 3:**
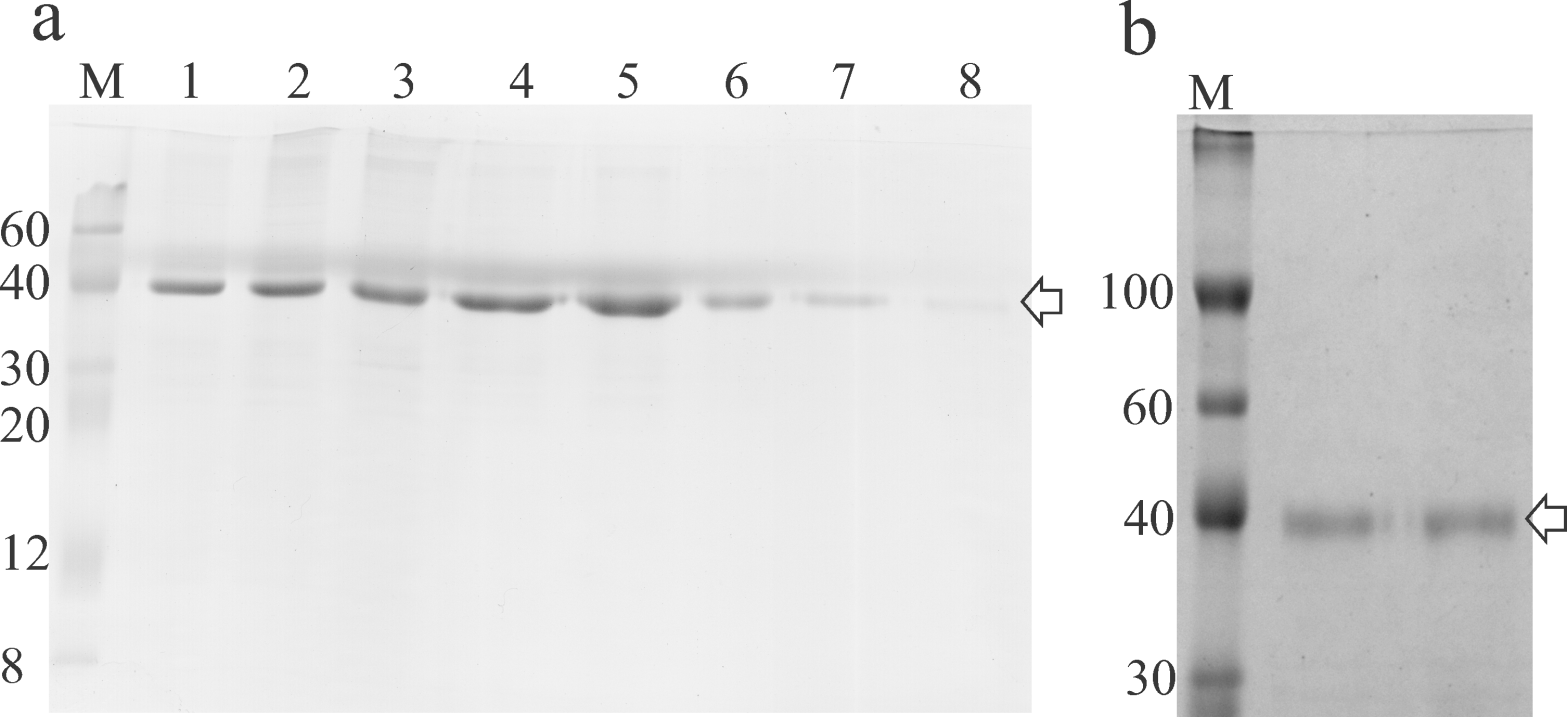
SDS-PAGE of samples produced during purification and refolding of B18R. Panel A. Lanes: 1, a solution of inclusion bodies in an SLS-containing buffer; 2, a solution after 20-hour incubation in a presence of CuSO_4_ for oxidation; 3, a sample of B18R after precipitation to remove SLS, dissolved in buffer A with 6M urea; 4–8, fractions (1 ml) collected during elution of B18R from an IMAC column. M—protein Mw marker (Sigma Cat. C1992), molecular masses of the marker’s bands indicated. Panel B. Results of ion exchange chromatography. Two lanes beside a marker’s lane were loaded with 10 ug of purified B18R. Arrow points at a band of B18R (Mw 38.9 kDa).

It was found that the recombinant protein is produced in high amounts in bacterial cultures of the producing strain induced with 1 mM IPTG, although the entire expression product accumulates in inclusion bodies. As proteins in inclusion bodies do not possess natural 3D structures and have no biological activity there was a need to develop a procedure for refolding of B18R. We tried to dissolve the inclusion bodies using various solubilizing agents (urea, guanidine hydrochloride, sodium lauroyl sarcosinate (SLS)), and found that SLS is most compatible with oxidative refolding, because in a series of experiments solubilization with SLS stably resulted in obtaining of the soluble biologically active protein. Results of a purification of B18R are shown in Fig 3. The calculated Mw of recombinant B18R is 38.9 kDa.

To investigate other factors which contribute to an efficiency of solubilization, various pH values of the solubilization buffers were tried. All solubilization buffers contained 2% SLS. At pH 8 a pellet of inclusion bodies did not dissolve completely even during long (20 hrs) incubations although it was possible to purify soluble B18R from the solution. At pH 10 or pH 12 the pellet dissolved immediately and the solution remained clear through further steps of the purification.

A first step of refolding of B18R was oxidation of free sulfhydryl (-SH) groups to form disulfide bridges within the protein globule. Oxidation was done at pH 10 by exposing the SLS-solubilized protein to air in a presence of copper ions (Cu^2+^) for long time. A protein purification was achieved using a combination of two chromatographic methods.

The protein was precipitated with (NH_4_)_2_SO_4_ to remove bulk of SLS which remained in a solution. Recombinant B18R carries an N-terminal His-tag used to adsorb the protein onto an IMAC column. The protein pellet was dissolved in 6M urea which helps dissociation of the remaining detergent from the protein. It was found that the addition of urea (6M) is required for the protein to bind to the column. In a separate experiment, an attempt was made to load completely refolded and purified B18R onto an IMAC column without a presence of any denaturing agents. In this attempt it was found that N-terminally His-tagged B18R remains in flowthrough. A complete removal of detergents was achieved by extensive dialysis of the IMAC product. SLS is the dialyzable detergent because it has a high critical micelle concentration (CMC 0.42%). A second round of purification was done using a different type of chromatography (AEX). An isoelectric point (pI) of B18R is 6.40. The protein adsorbed to Q-sepharose which is the anion exchanger at pH9.0 and eluted with 1M NaCl. Dialysis was used to exchange solutions after the IMAC and AEX, and no protein precipitation was observed at either dialysis. In a series of experiments, inclusion bodies in amount of >700 mg (wet pellet) were obtained from 1L-cultures. Upon complete processing of a frozen portion of inclusion bodies (70 mg) typical yields of soluble B18R were 35–40 mg. The resulting protein is >95% pure (Fig 3B). A residual endotoxin contamination in the final preparation was tested with the LAL test (Endosafe-PTS) and is <5 EU/ml. When recombinant B18R was added (to 500 ng/ml) to cultures BHK-21 or HEK293 no visible cytotoxicity was observed during 7-day incubations.

### Analytical size exclusion chromatography

**Fig 4:**
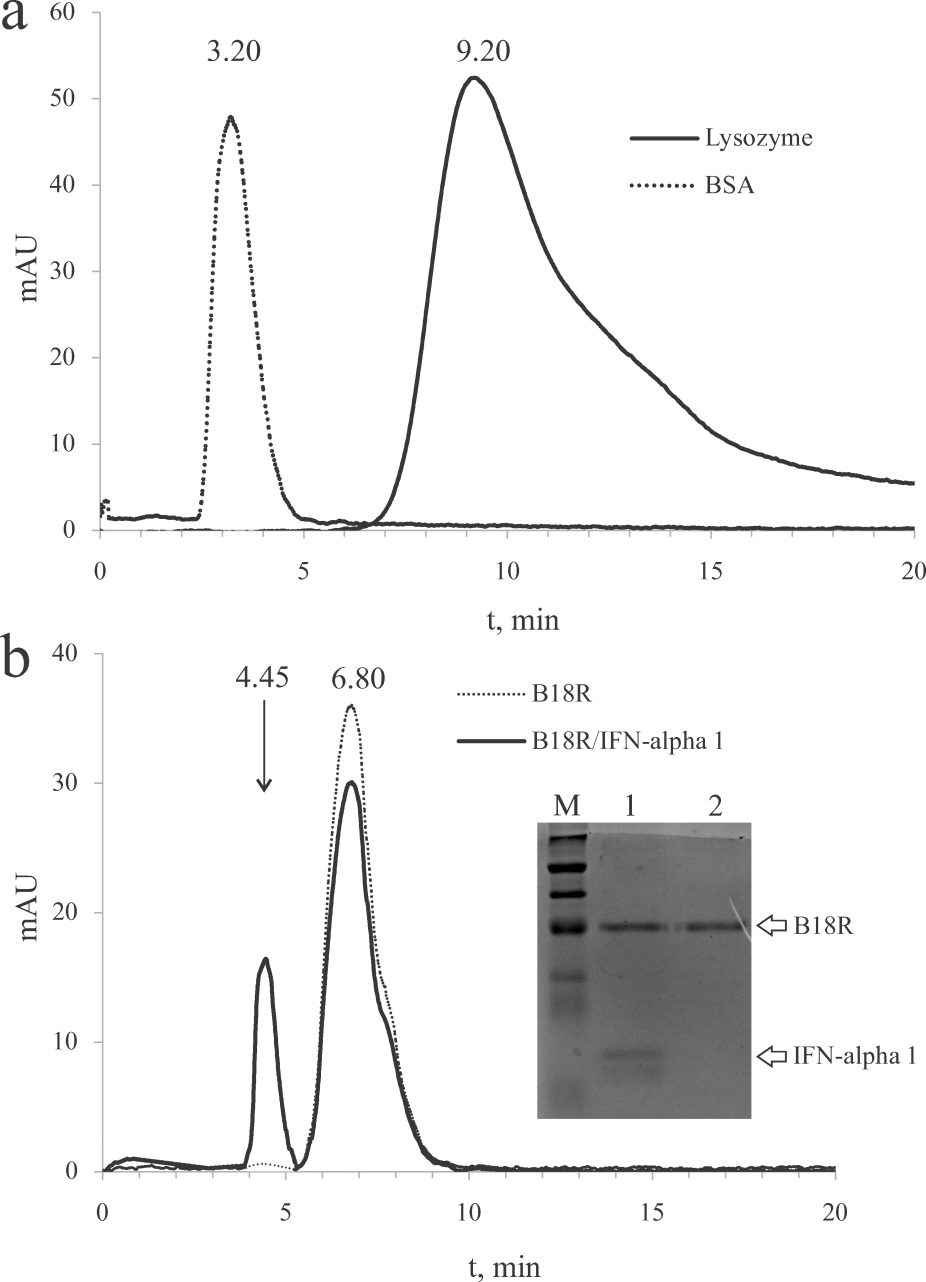
Analytical gel filtration of B18R and its complex with IFN-alpha 1. Panel a, lysozyme and BSA were run through a SEC column to obtain reference retention times. Retention times are shown above corresponding peaks. Panel b, B18R migrates during the SEC as a monomer (retention time 6.80 min). In a mixture of B18R with IFN-alpha a complex is formed which retention time (4.45 min) indicates that a stoichiometry of the complex is 1:1. Inset in panel b: fragment of an SDS-PAGE gel on which a material was loaded from the peaks at 4.45 min (lane 1) and 6.80 min (lane 2).

Results of an analytical SEC are shown in Fig 4. The SEC column was calibrated by determining retention times for lysozyme (Mw 14.3 kDa) and bovine serum albumin (BSA; Mw ~66 kDa). The obtained retention times (Fig 4A) allow to determine a form in which B18R is present in a solution and also calculate a stoichiometry of a B19R/IFN-alpha 1 complex. B18R has the retention time intermediate between those for lysozyme and BSA indicating that B18R is a monomer. B19R was mixed with IFN-alpha 1 (Mw 19.4 kDa) in a molar ratio of 4.5:1 and the resulting mixture was analyzed by the SEC. The SEC results (Fig 4B) show an appearance of two peaks; the retention time of the first peak (4.45 min) is less than that of free B18R, and of the second peak (6.80 min) is the same as for B18R. The first peak corresponds to the B19R/IFN-alpha 1 complex as was evidenced by SDS-PAGE of the chromatographic peaks (inset in Fig 4B). The second peak contains B18R. The retention time of the first peak is indicative of a 1:1 stoichiometry in the B19R/IFN-alpha 1 complex. An amount of B18R in the complex may be assessed by a comparison of the peaks’ areas. The area was estimated as a product of the peak height and width at half-height. According to the estimates, the second peak of the mixture B19R+IFN-alpha 1 contains ~79% of an expected peak if all B18R is free. Given a total amount of 190 ug of B18R in the mixture, 39.9 ug of B18R is bound in the complex which amount perfectly matches the amount (20 ug) of IFN-alpha 1 in the mixture. Thus the refolded B18R binds to IFN-alpha and supposedly is capable of neutralization of the type I IFNs in cell cultures.

### VEE replicons

We constructed autonomously replicating fragments (replicons) of a genome of *Venezuelan equine encephalitis virus* (VEE) for use in an investigation of an ability of recombinant B18R to support a replication of the RNA-vector with known sensitivity to the antiviral state. The replicons were engineered to produce either GFP (into a cytoplasm) or G-CSF (into a culture medium). The alphavirus replicons were chosen for this work because high levels of a protein expression impart them with prospects for use in a large-scale production of proteins with high added values, e.g. biopharmaceuticals.

One important feature of the alphavirus replication is its profound cytopathic effect which is typical for all wild-type strains of alphaviruses. The cytopathicity presents as a rapid die-off of transfected culture and precludes a development of long-lasting producer lines. In a search for efficient vectors which do not kill producer cells a number of mutations have been identified reducing the cytopathicity of alphaviruses. Various mutations localized in genes encoding non-structural proteins (nsP) allow the noncytopathic replication of SIN replicons [31], SFV replicons [32] and VEE replicons [30]. Authors incorporated the mutation described in [30] into the replicon which contains a replicative machinery from TC-83 strain and has no genes for structural proteins. The obtained replicons caused non-cytopathic infections in BHK-21 and HEK293 cells.

The replicon’s cDNA-copy was produced by a ligation of several fragments which were assembled *de novo* and sequencing-verified. A GFP gene was cloned into a RepVEE-GFP replicon under control of the VEE subgenomic promoter. A genetic map of the replicon RepVEE-GFP is depicted in Fig 2.

**Fig 5:**
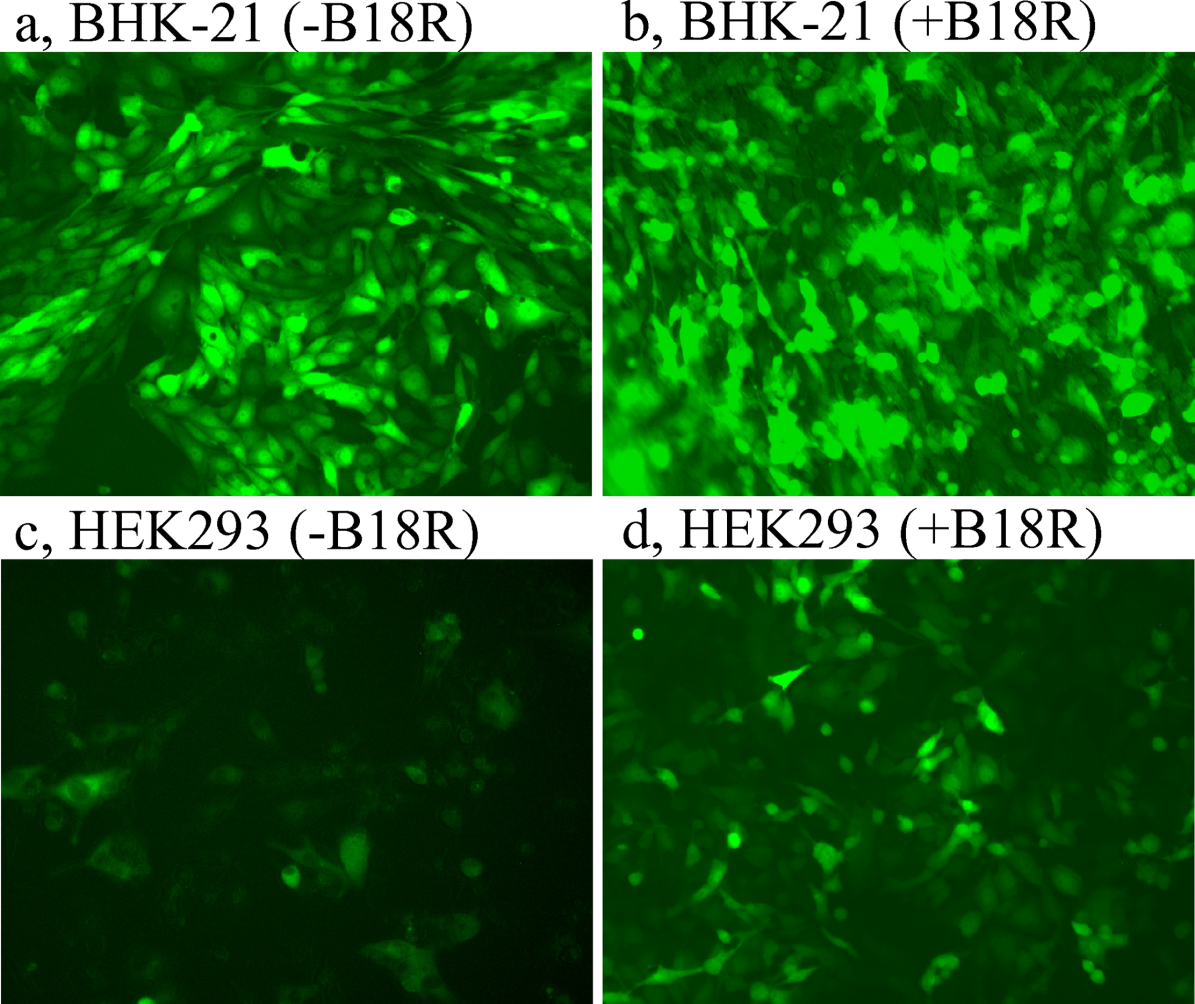
GFP fluorescence in cultures transfected with the replicon RepVEE-GFP, 24 hrs after transfection. Panels: a, BHK-21, no B18R; b, BHK-21, with B18R; c, HEK293, no B18R; d, HEK293 with B18R.

RNA of the replicon RepVEE-GFP was transfected into BHK-21 or HEK293 cells, and immediately after the transfection the cells were split into several flasks. Recombinant B18R was added immediately after the transfection to a half of the cultures to 500 ng/ml, another half of the cultures (without the addition of B18R) served as controls. Examination of the BHK-21 cultures at 24 hrs after the transfection revealed that regardless of the presence of B18R ~90% of cells demonstrated GFP fluorescence (Fig 5A and 5B). Fractions of the GFP-positive cells in the BHK-21 cultures were similar over duration of the experiment regardless of the presence of B18R.

On the other hand, the transfected cultures HEK293 demonstrated a higher intensity of fluorescence in the presence of B18R (Fig 5C and 5D). In a separate experiment, B18R was added to HEK293 cultures transfected with RepVEE-GFP to various final concentrations: 500, 200, 100 and 20 ng/ml. Intensities of GFP fluorescence were similar in the cultures with 500 and 200 ng/ml B18R, and in both cultures were all along higher than in cultures with 100 and 20 ng/ml B18R, during a 5-day experiment. At day 5 after the transfection the culture with 20 ng/ml B18R was similar to the culture without the addition of B18R and both cultures consisted from GFP-negative cells. Addition of B18R to the high concentration (500 ng/ml) to naïve HEK293 cells did not change morphology of the cells or a growth rate of the culture and thus recombinant B18R is not toxic to the cells. Having an abundance of bacterially expressed B18R, the protein was used at the high concentration in further experiments.

### B18R helps expression from a RNA replicon in HEK293 cells

A replicon RepVEE-Pac-2A-SigP-GCSF was created by replacing the GFP gene in the replicon pRepVEE-GFP with a gene cassette Pac-2A-SigP-GCSF. The gene cassette encodes a polyprotein which is cotranslationally cleaved into two parts because of a presence of the autoprotease FMDV 2A within the polyprotein. The FMDV 2A interacts with a translating ribosome in such a way so that the ribosome does not form a peptide bond between the last two residues (Gly-Pro) within the FMDV 2A sequence, although the ribosome does not dissociate from an mRNA (instead the ribosome continues translating a remaining part of the polyprotein). As a result, two proteins are generated (Pac-2A and SigP-GSCF), one of which (Pac-2A) harbors the activity of the puromycin acetyltransferase and inactivates puromycin. The protein SigP-GCSF is a precursor protein for G-CSF which signal peptide (SigP) directs the protein to enter a cell’s secretory pathway. Ultimately SigP is cleaved off and G-CSF undergoes glycosylation and subsequently released out of cells. It appears that despite SigP carries an additional residue (proline) at the N-end it does not hamper the function of SigP.

Incubation of cultures in a medium with puromycin allows selecting of a subpopulation of cells chronically infected with the replicon, provided that the replicon itself is capable of persistence in the used cell lines. In initial experiments authors tried to obtain cultures stably producing G-CSF by subjecting of the transfected cultures BHK-21 and HEK293 to selection with puromycin. No inhibitor of IFNs was added to the cultures in the initial experiments. Puromycin was added 24 hrs after the transfection. Control (mock-transfected) cultures BHK-21 or HEK293 rapidly died after the addition of puromycin (results not shown). The replicon-transfected culture BHK-21 passed through an initial period of acute infection (~36 hrs) after which the cells resumed growth in a medium with puromycin and the established culture was similar in morphology and growth rate to the naive BHK-21 cells. This culture is further referred to as BHK21rep. In contrast, the transfected culture HEK293 terminated its growth in a presence of puromycin. The latter culture was abandoned and an experiment with the transfection was repeated with an addition of B18R.

**Fig 6:**
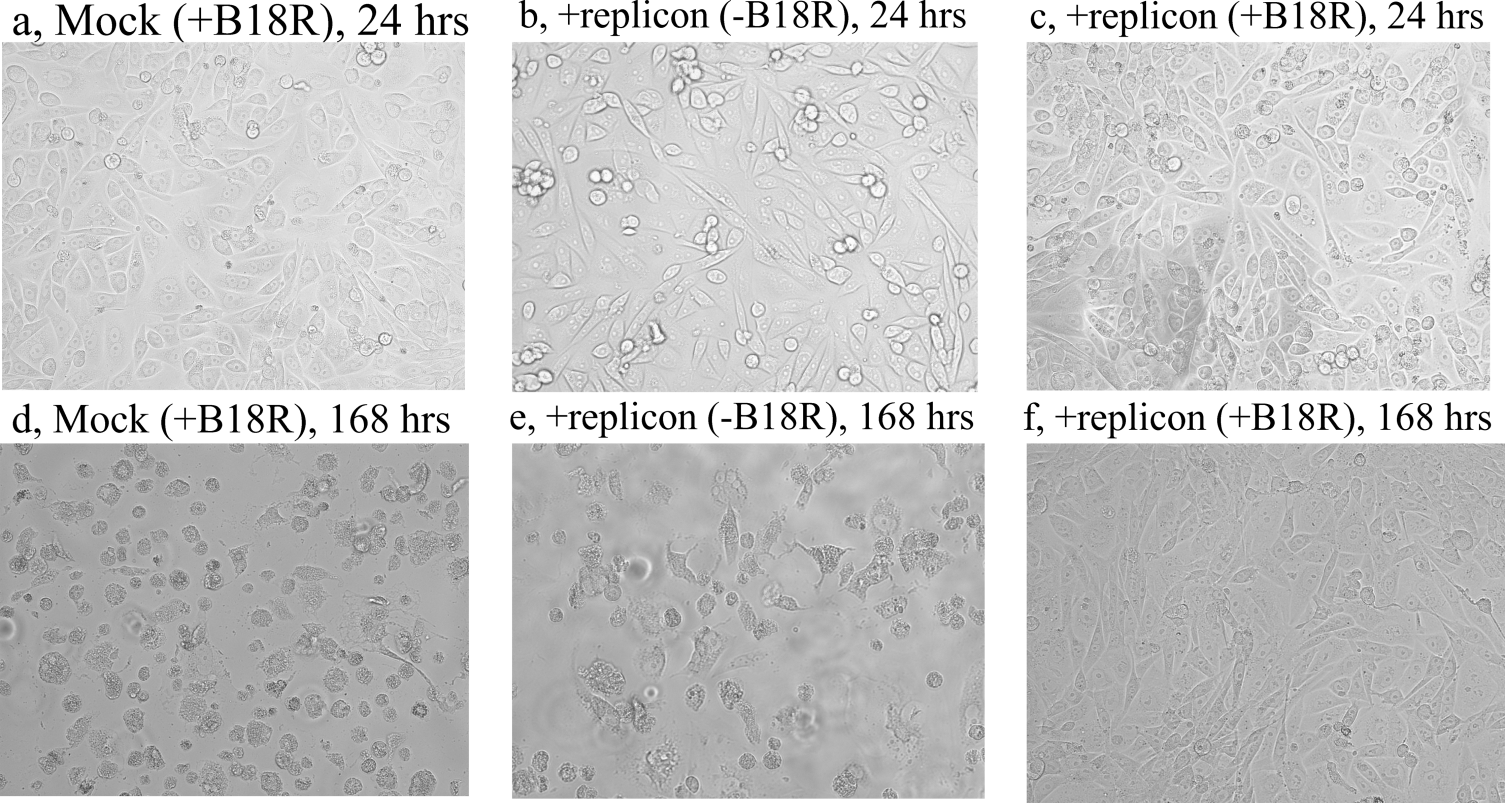
B18R enables persistence of the VEE replicon in HEK293 culture. Time after transfections indicated in captions above panels. Puromycin was added to all cultures 24 hrs after the transfections.

Upon transfecting of HEK293 with RNA of the replicon RepVEE-Pac-2A-SigP-GCSF the transfected culture was split into several flasks. Recombinant B18R was added to one half of the flasks immediately after the transfection to a final concentration 500 ng/ml. To the other half of the transfected culture no inhibitor of IFNs was added. B18R was also added to the control (mock-transfected) HEK293 culture. A selection with puromycin started at 24 hrs after the transfections. The mock-transfected culture died within 24 hrs upon the addition of puromycin because of a high concentration of the antibiotic (Fig 6A and 6D).

The replicon-transfected culture HEK293 maintained in a medium with puromycin but without B18R also died (Fig 6B and 6E). The other half of the same culture which was grown in a presence of B18R remained alive during a 7-day incubation in the presence of puromycin (Fig 6C and 6F). The latter cells (designated HEK293rep) were expanded and cryo-preserved. During subsequent experiments this culture was recovered from the cryo-stock and propagated in a medium containing puromycin and B18R.

The cultures BHK21rep and HEK293rep were propagated to 90% confluency in T150 flasks in a presence of puromycin and B18R. From this point the cultures were incubated in a production medium which contains 1% FBS and does not contain puromycin. The content of the serum (FBS) was lowered compared to the complete medium to slow down a growth rate of the cultures because during a 7-day experiment the cultures tend to die from overconfluence in incubated in the complete medium. The cultures to which B18R was added (500 ng/ml) were designated “+B18R”; the IFN inhibitor was not added to the cultures designated “–B18R”. An accumulation of G-CSF in the production media at various time points was measured using a quantitative ELISA.

**Fig 7:**
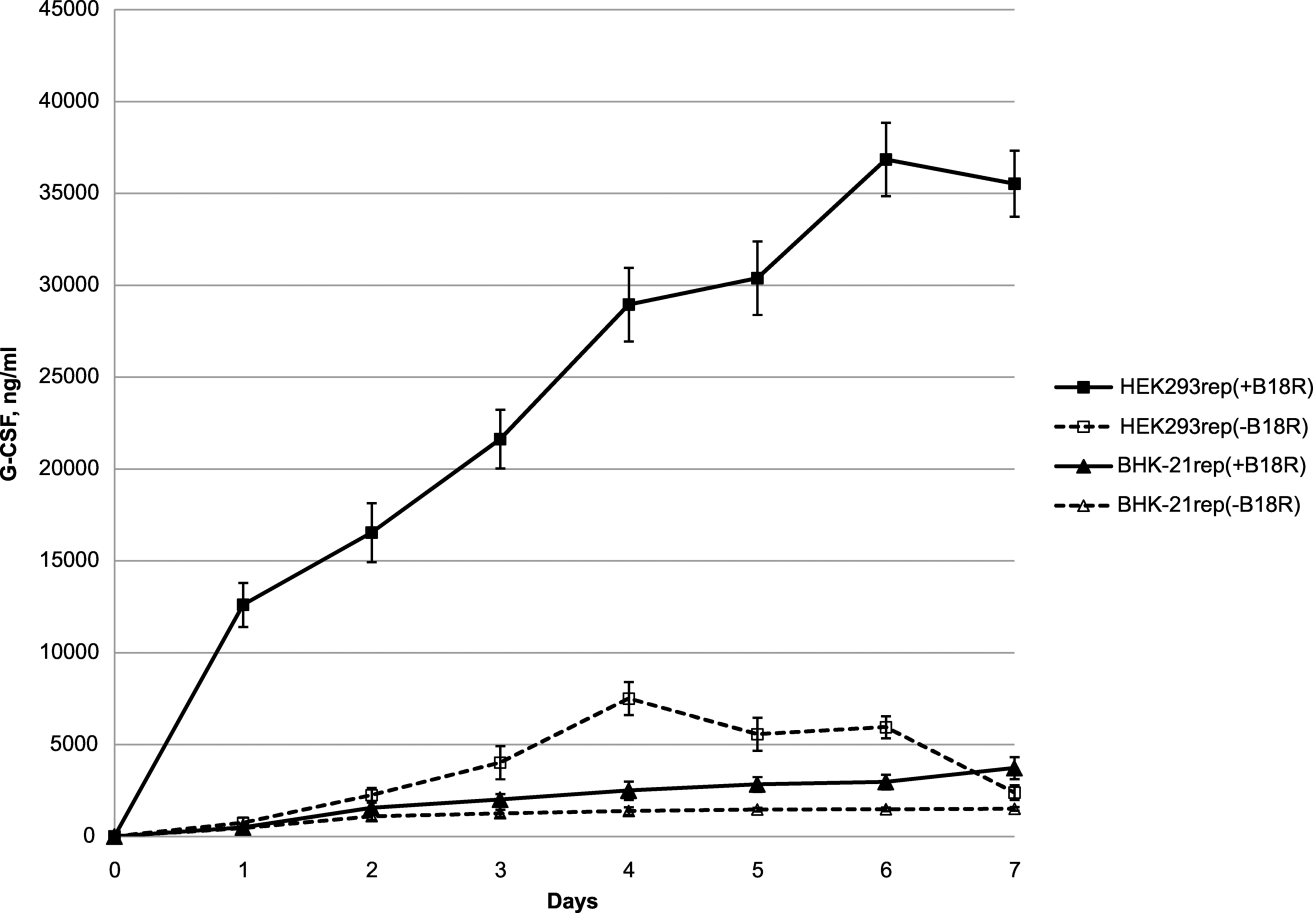
Accumulation of G-CSF in incubation media of HEK293 and BHK21 cultures transfected with a G-CSF-producing VEE replicon. Data for two producing cultures based on BHK-21 or HEK293 are shown. A presence of B18R in incubation media significantly increases yields of the product of recombinant expression (G-CSF) driven by the VEE replicon.

The cultures BHK21rep provided similar levels of the accumulation of G-CSF regardless of the addition of B18R (3.7 μg/ml at day 7 for “+B18R”; 1.5 μg/ml for “–B18R”) (Fig 7). In the HEK293-based culture infected with the producing replicon, the production was considerably higher and the presence of B18R significantly increased the product yields (Fig 7). The highest concentrations of G-CSF in the collected samples were 35.5 μg/ml for “+B18R” (at day 7), 7.5 μg/ml for “–B18R” (at day 4, subsequent concentrations diminished).

## Discussion

The protein B18R is a type I IFNs inhibitor and is used in a variety of cell technologies. Its currently most important field of application is a cellular reprogramming in which B18R allows utilization of RNA-vectors, e.g. synthetic mRNAs or replicons of RNA-viruses [11, 24, 25, 33]. A wide field of possible applications would have incurred intensive studies and an availability of this protein, although only one paper [28] describes obtaining of bacterially expressed B18R. Still a crystal structure is not available for B18R and no anti-B18R antibodies are present on a market.

Considering a deficiency of information on obtaining of bacterially-expressed B18R authors elected to produce recombinant B18R in *E*.*coli*. The only previously described method [28] uses an extraction with 6M guanidine and refolding in a presence of 0.7M arginine and a glutathione redox pair. The published method is not easy to reproduce and in preliminary experiments authors failed to produce soluble B18R using the above protocol because the protein precipitated when guanidine was removed by dialysis.

During the expression in *E*.*coli* B18R goes into inclusion bodies. A new method for refolding of B18R extracted from the inclusion bodies was developed. The inclusion bodies are dissolved in a buffer containing a mild detergent (SLS) and the denatured protein in solution is oxidized. Then the detergent is removed using a precipitation and chromatography in a presence of urea which keeps the protein in a solution. It was found that removal of copper ions from the solution before the IMAC is needed; otherwise His-tagged B18R goes into flowthrough presumably because the His-tags are occupied with copper. The whole procedure provides several hundred milligrams of soluble and biologically active B18R from 1 L of an induced bacterial culture. The resulting protein has <5 units/ml of an endotoxin contamination, and at a concentration 500 ng/ml it is safe for tested cell cultures.

It was demonstrated that when B18R is added to a culture medium, HEK293 cells maintain a RNA replicon and B18R increases a production of a target protein encoded in the RNA-replicon.

Members of various families of RNA-viruses attract attention for a use as vectors for an expression of recombinant proteins in eukaryotic cells. For example, replication of engineered alphaviruses leads to a synthesis of large amounts of heterologous proteins from genes placed into the replicating genomes [34, 35, 36]. Alphaviruses have a broad host range and can replicate in a variety of cell types, including nondividing cells, from mammalian or avian species and mosquitoes. Although, an obstacle exists which limits a scope of a possible utilization of the alphavirus vectors in the expression systems, which is sensitivity of these vectors to antiviral effects of type I IFNs [37, 38, 39, 40, 41].

With a background of the use of the alphavirus vectors for large-scale protein expression, gene delivery *in vivo*, and as replicative backbones for live vaccines, there is one field of the utilization in which the alphavirus’ replicons are exceptionally well suited. This is an epigenetic reprogramming employing RNA-vectors to produce medical-grade cell products. A development of the cell products such as induced pluripotent stem cells (IPSCs) using the epigenetic reprogramming requires an expression in primary cultures of a set of regulatory proteins (reprogramming factors) in high amounts and for a long time. If the reprogrammed cells are to be used in medicine, all vector-specific elements must be completely removed, thus precluding a use of retroviruses and lentiviruses which were the first viral vectors for the reprogramming. The alphavirus vectors are different in a fashion that they can be completely removed from the cells because they do not integrate any sequences into host’s genome. The alphavirus vectors showed an exceptionally high efficiency in the process of producing of the IPSCs [11]. Importantly the resulting IPSCs are autologous and syngenic to a patient’s body thus complying with the medical requirements. Although the primary cultures rapidly develop the antiviral state which ceases an infection with the VEE replicons in just 1 day if no inhibitor of IFNs is used [11]. The persistent infection with RNA-vectors may be achieved by an addition of the IFN-inhibitors such as B18R. In majority of studies conditioned media (CM) which contain the inhibitor. These CM are collected from cell cultures infected with *Vaccinia virus* or transfected with a B18R-producing plasmid. Amounts of B18R in the CM are generally not controlled, thus an efficiency of the reprogramming may be suboptimal. In turn, B18R available from commercial vendors is universally a product of expression in insect cells infected with a recombinant baculovirus and the product is expensive. The herein protocol provides better controlling of components in reprogramming cocktails and other processes which require inhibition of IFNs compared to the use of CM and also it is a disparately more economic alternative to baculovirus-expressed B18R.

HEK293 is a cell line widely used (including in industrial settings) for an expression of recombinant proteins [42]. This cell line is capable of limiting a replication of RNA viruses by invoking the IFN response and through a mechanism of RNA interference (RNAi) [43, 44]. In an expression system utilizing HEK293 and a RNA-vector, when B18R was added to the producing cultures, a yield of a product increased 4.7 times. This result underscores an advantage of using of bacterially-expressed B18R in RNA-vector-based mammalian expression systems.

## Supporting information

S1 File Detailed description of methods. Description of methods and strategies not covered in the main text.
(DOCX)

S2 File Chromatograms from an analytical SEC. Chromatographic curves exported to excel.
(XLSX)

S3 File ELISA Calibration curve. A calibration curve produced using a standard G-CSF sample from a quantitative ELISA kit.
(XLSX)

S4 File ELISA data. OD values in wells and calculated G-CSF concentrations.
(XLSX)
