# Supplementary material for: Recombinant Vaccinia virus-coded interferon inhibitor B18R: Expression, refolding and a use in a mammalian expression system with a RNA-vector
Source: PLoS One. 2017 Dec 7;12(12):e0189308. doi: 10.1371/journal.pone.0189308 (PMC5720773; doi:10.1371/journal.pone.0189308)
Supplement: S1 File — Description of methods and strategies not covered in the main text. (DOCX) [file pone.0189308.s001.docx]

Recombinant Vaccinia virus interferon inhibitor B18R: expression, refolding and a use in a mammalian expression system with a RNA-vector

Supporting information

**Synthesis of DNA fragments**

Synthetic gene B18R and G-CSF and fragments to assemble a VEE replicon were produced *de novo* using an overlap extension PCR. At a first step a set of oligonucleotides was devised using a service “Assembly PCR Oligo Maker” at the York University (<http://www.yorku.ca/pjohnson/AssemblyPCRoligomaker.html>). This allows calculating sets of long oligonucleotides which together cover sequences of the fragments to be synthesized. The primers alternate as "sense"->"antisense"->"sense"-> etc. Pairs of the adjacent primers overlap with an annealing temperature 60°C. The calculated primers are designated as "inner" primers. A pair of "flanking" primers was used which target positions at ends of a fragment. The flanking primers incorporate restriction sites.

The overlap extension PCR is performed in two rounds. On the 1^st^ round PCR is performed with a mixture of all "inner" primers. No exogenous template is added. The 1^st^ round results in a generation of products of stochastic synthesis of DNA which also contain a nearly full-length fragment of interest. Then the 2^nd^ round PCR is performed with the flanking primers. Results of the 2^nd^ round PCR were separated by electrophoresis in 1% agarose. The fragment of an expected length was excised and cloned into the pGEM-T vector (Promega). Inserts in selected clones were sequenced for confirmation that they contain no PCR-introduced errors.

**Cloning strategy to assemble VEE replicons**

An autonomously replicating fragment of genome (replicon) of Venezuelan equine encephalitis virus (VEE) was produced by performing several stages of an assembly from the synthetic DNA fragments (listed in Table S1). A sequence of the replicon corresponds to the Genbank entry No L01443 (VEE strain TC-83). The replicon also has one mutation which makes replication noncytopathic.

**Table S1. Synthetic DNA fragments to assemble a VEE replicon.**

| Fragment | Flanking restriction sites^a^ | Length^b^, bp |
| --- | --- | --- |
| 5UTR^c^ | AasI (vector) - BspEI (312) | 396 |
| A | BspEI (312) - SalI (1620) | 1309 |
| B | SalI (1620) - BlpI (3748) | 2127 |
| C^d^ | BlpI (3748) - NheI (5328) | 1581 |
| D | NheI (5328) - AclI (6417) | 1089 |
| E | AclI (6417) - PspOMI (7501) | 1084 |

Notes: ^a^ numbering of restriction sites’ positions is given with the reference to the Genbank entry #L01443; ^b^ length of a VEE-specific fragment which is excised by digestion with indicated restrictases; ^c^ synthetic fragment 5UTR contains a VEE-specific sequence from the first nucleotide in a VEE genome (A) to the first site BspEI. Upstream of the VEE-specific sequence an SP6 promoter is placed and there is a synthetic linker which terminates with AasI site; ^d^ the synthetic fragment C is identical to a homologous fragment in the Genbank entry L01443 except that timidine in position 3865 is replaced with adenine, this mutation changes the encoded aminoacid from Gln to Leu.

The *de novo*-constructed fragments are devised so that they are flanked with restriction sites which are rare in a VEE genome. Results of restriction analysis of clones are shown in Fig S1.


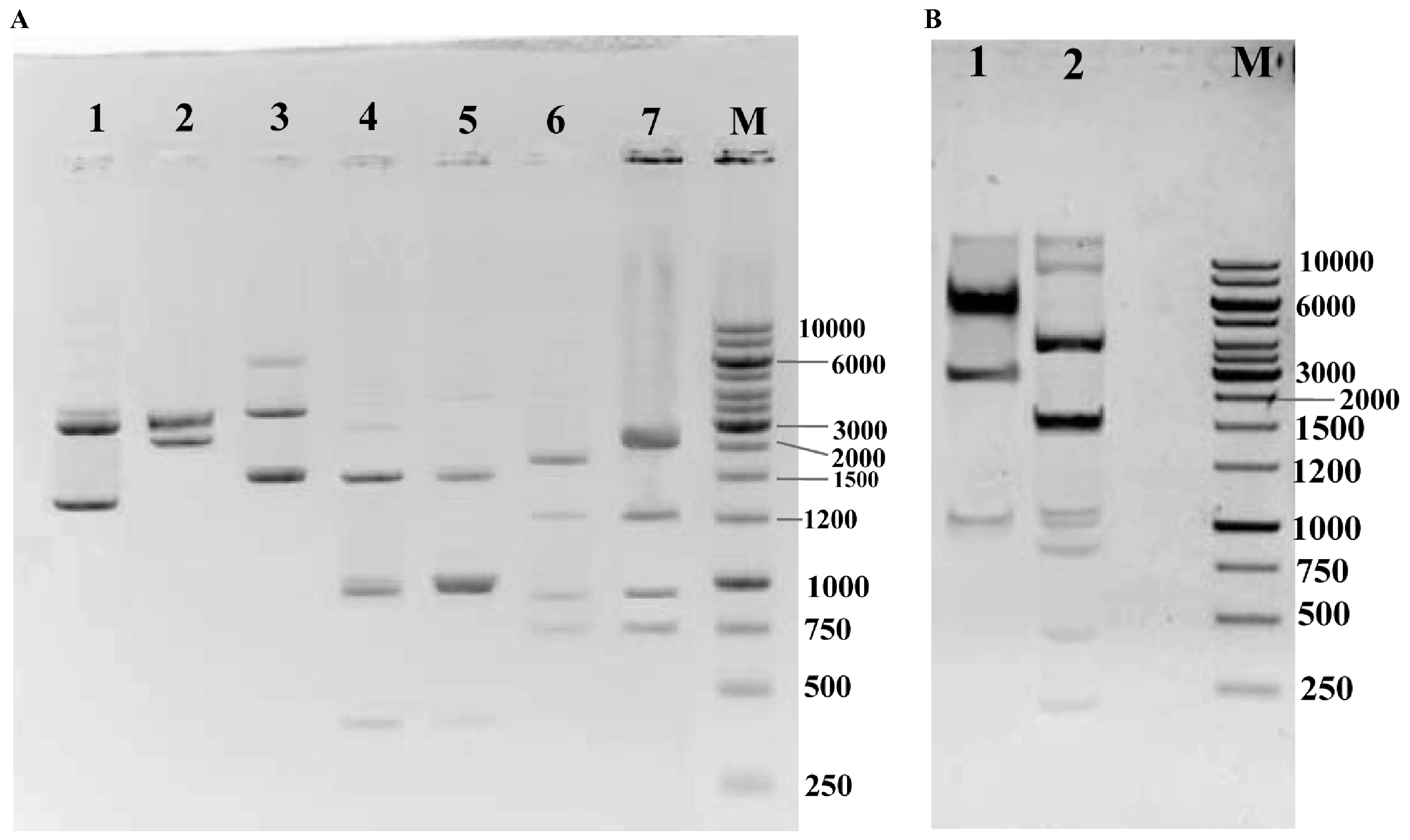


**Fig. S1. Restriction analysis of plasmids containing fragments of a VEE genome.** Panel a, lane 1, digestion of plasmid pGEM-T(A) with restriction enzymes BspEI+SalI generates three fragments: 2982 bp, 1309 bp and 34 bp; lane 2, digestion of pGEM-T(B) with SalI+BlpI (fragments: 2982, 2127, 34 bp); lane 3, plasmid pGEM-T(C) with BlpI+NheI (fragments: 3017, 1581 bp); lane 4, plasmid pGEM-T(D) with NheI+AclI (fragments: 1579, 1089, 1063, 373 bp); lane 5, plasmid pGEM-T(E) with AclI+PspOMI (fragments: 1580, 1084, 1016, 373, 48 bp); lanes 6-7, digestions of irrelevant plasmids. Lane M - O'GeneRuler 1kb DNA Ladder (Fermentas). Panel b, lane 1, digestion of plasmid pB-E with a pair of restrictases SalI+PspOMI produces four fragments: 5881, 2935, 1027 and 46 bp; lane 2, digestion of pB-E with AclI+NheI (fragments: 4770, 1578, 1089, 1029, 817, 373 and 233 bp). Lane M - O'GeneRuler 1kb.

The fragments B (SalI-BlpI), C (BlpI-NheI), D (NheI-AclI) and E (AclI-PspOMI) were combined in a ligation mixture with a vector produced from a derivative of pUC18 which has cloning sites SalI and PspOMI. Upon transformation of *E.coli* with the ligation mixture a plasmid was selected which contains 5881 bp of the VEE-specific sequence. This plasmid was designated pB-E.

The fragment 5UTR contains VEE’s 5’-untranslated region (5’-UTR) and a part of the first ORF in the VEE genome (from a starting codon ATG to BspEI site). A sequence of a promoter for SP6 RNA polymerase (ATTTAGGTGACACTATAG) was placed immediately upstream of the first VEE-specific nucleotide in the fragment 5UTR. The SP6 promoter allows producing a replicon RNA using *in vitro* transcription. Also the fragment 5UTR contains a linker which terminates with a restriction site AasI which was used for insertion of this fragment into an ultimate plasmid. In a separate ligation reaction a ligation mixture contained the fragment 5UTR (396 bp) (excised using AasI+BspEI), the fragment BspEI-SalI from pGEM-T(A) (1309 bp), the fragment AatII+AasI from pUC18 (162 bp) and a vector pUC18|AatII+SalI (2204 bp). Transformation of *E.coli* with the latter ligation mixture and selection of clones resulted in obtaining of a plasmid pUTR-Sal, which carries 1625 bp of the VEE-specific sequence (from the first nucleotide to a SalI site).

The plasmids pB-E and pUTR-Sal together comprise a nearly full-length first ORF in the VEE genome. The first ORF encodes nonstructural proteins (nSP) which are components of a replication complex. A restriction site PspOMI which terminates the VEE-specific sequence in the pB-E can be considered as a natural upstream border of the subgenomic promoter (SP). The SP is a region in the VEE RNA (actually in the negative-polarity “antigenomic” RNA) from which synthesis of subgenomic RNA (sgRNA) is initiated. Preservation of the SP and adjacent sgRNA 5’-UTR is needed to express genes of interest. The genes of interest were positioned downstream of the SP in place of genes for structural proteins.

During a next step of the assembly, sequences of VEE 3'-UTR and SP were produced. Gene encoding green fluorescent protein (GFP) was amplified from an existing plasmid in laboratory’s collection. At this stage, sequences of the VEE SP and sgRNA 5’-UTR were added upstream of the GFP gene by using extended primers. To construct a 3’-end of the replicon, the VEE 3’-UTR was added downstream of the GFP gene. The sequence added downstream of the GFP gene includes nucleotides from a last site MfeI to the last C residue in Genbank entry #L01443 (…ATTTC), followed by a stretch of adenine residues (A_25_) (this oligo-A stretch is to increase a stability of the replicon RNA in cells) and a restriction site MluI. The MluI site is unique in an ultimate construct and is used to linearize the plasmid DNA before transcription. The resulting fragment was cloned into the pGEM-T vector to produce a plasmid p3UTR. The fragment PspOMI+MluI (943 bp) was excised from the p3UTR plasmid and ligated with the fragment SalI+PspOMI from pB-E (5881 bp), the fragment AatII+SalI from pUTR-Sal (1867 bp) and a vector obtained from pGEM-T by digestion with AatII+MluI (2921 bp). The resulting construct is named a plasmid pRepVEE-GFP. The plasmid pRepVEE-GFP encodes the VEE replicon which is capable of expressing GFP during intracellular replication.

To produce a replicon which drives an expression of recombinant human granulocyte colony stimulating factor (G-CSF), the GFP gene in the pRepVEE-GFP replicon was replaced with a gene cassette Pac-2A-SigP-GCSF. The gene cassette Pac-2A-SigP-GCSF directs expression of two proteins of which one protein has activity of puromycin N-acetyltransferase (Pac) and the other is G-CSF. The gene cassette Pac-2A-SigP-GCSF has one long ORF which encodes a polyprotein wherein N-terminal part is Pac, C-terminal part is G-CSF and a central region encodes foot-and-mouth disease virus (FMDV) protease 2A. FMDV 2A is referred to as “autoprotease” because its biological action results in separation of the polyprotein into two parts. In reality FMDV 2A interacts with a translating mammalian ribosome so that the ribosome does not form a peptide bond between the last two residues Gly-Pro in FMDV 2A. This does not lead to dissociation of the ribosome from mRNA; rather the ribosome continues translation of the polyprotein. Two proteins are generated as a result, one protein is Pac-2A and the other is SigP-GSCF. The protein Pac-2A incurs an activity of the enzyme which inactivates antibiotic puromycin. The protein SigP-GCSF is a preprotein of G-CSF (contains a signal peptide which directs G-CSF into a cell secretory pathway). A sequence of the gene cassette Pac-2A-SigP-GCSF is depicted in Fig S2.


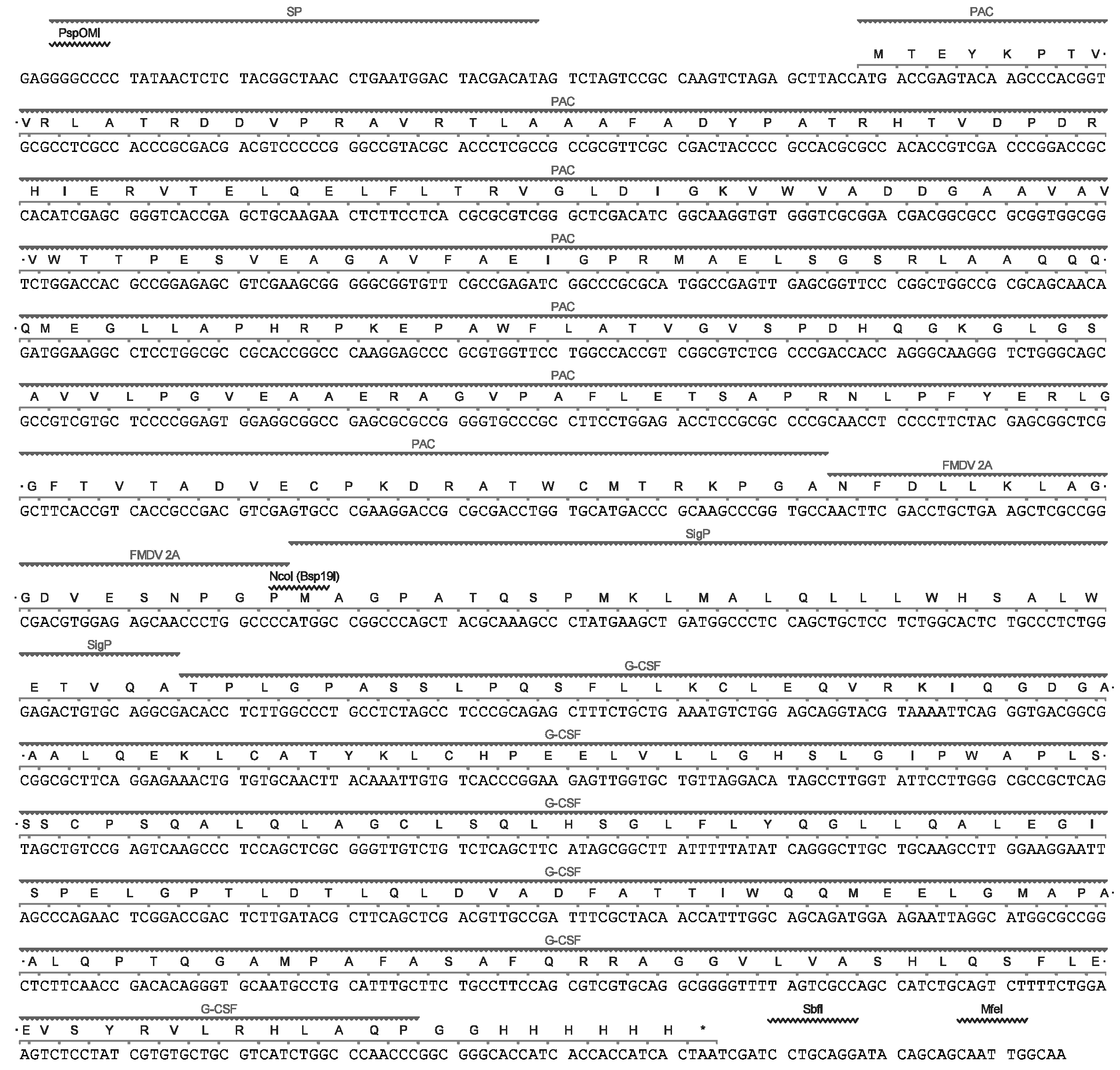


**Fig. S2. Gene cassette Pac-2A-SigP-GCSF.** Deduced translation of an encoded polyprotein is given above the nucleotide sequence. Individual functional proteins are marked by notched lines. Restriction sites used for an assembly of the gene cassette are depicted.

**Quantitative ELISA**

An ELISA was performed with a use of Human G-CSF/CSF3 ELISA Kit (Sigma Cat# RAB0103). To determine amounts of G-CSF in cultural media, serial dilutions were made from each sample to cover a range of dilutions 1:100-1:1,000,000. Dilutions of a standard solution of G-CSF (supplied in the kit) were prepared to cover a range of concentrations 0.69-500 pg/ml. The standards were used to build a calibration curve. G-CSF concentrations in experimental samples were calculated from the dilutions for which OD values fall into a linear range of the calibration curve.
